# Perturbed-Location Mechanism for Increased User-Location Privacy in Proximity Detection and Digital Contact-Tracing Applications

**DOI:** 10.3390/s22020687

**Published:** 2022-01-17

**Authors:** Elena Simona Lohan, Viktoriia Shubina, Dragoș Niculescu

**Affiliations:** 1 Electrical Engineering Unit, Tampere University, 33720 Tampere, Finland; elena-simona.lohan@tuni.fi; 2 Computer Science and Engineering Department, University Politehnica of Bucharest, 060042 Bucharest, Romania; dragos.niculescu@upb.ro

**Keywords:** location privacy, perturbation mechanism, proximity detection, digital contact tracing, multi-floor areas

## Abstract

Future social networks will rely heavily on sensing data collected from users’ mobile and wearable devices. A crucial component of such sensing will be the full or partial access to user’s location data, in order to enable various location-based and proximity-detection-based services. A timely example of such applications is the digital contact tracing in the context of infectious-disease control and management. Other proximity-detection-based applications include social networking, finding nearby friends, optimized shopping, or finding fast a point-of-interest in a commuting hall. Location information can enable a myriad of new services, among which we have proximity-detection services. Addressing efficiently the location privacy threats remains a major challenge in proximity-detection architectures. In this paper, we propose a location-perturbation mechanism in multi-floor buildings which highly protects the user location, while preserving very good proximity-detection capabilities. The proposed mechanism relies on the assumption that the users have full control of their location information and are able to get some floor-map information when entering a building of interest from a remote service provider. In addition, we assume that the devices own the functionality to adjust to the desired level of accuracy at which the users disclose their location to the service provider. Detailed simulation-based results are provided, based on multi-floor building scenarios with hotspot regions, and the tradeoff between privacy and utility is thoroughly investigated.

## 1. Introduction and Problem Statement

People are increasingly interconnected through their wireless devices, such as smartphones, smartwatches, and other wearable devices. Most of such devices are already capable of localization and sensing, either through Global Navigation Satellite Systems (GNSS) chipsets in outdoor scenarios or through IEEE802.11* (e.g., WiFi), Ultra-Wide Band (UWB), or Bluetooth Low Energy (BLE) chipsets in indoor scenarios. Many future wireless standards will also make localization and sensing as a part of the system design, such as emerging Sixth generation of cellular communications (6G) cellular communications [1], IEEE802.11bf WiFi upcoming standard [2], and UWB chipsets incorporated in modern smartphones [3].

Proximity-detection services based on wireless signals, and in particular based on BLE, have gained a significant interest in the past two years as they are enabling digital contract-tracing techniques [4] shown to be relevant in the context of COVID-19 disease management [5,6]. Magnetic-field proximity detection solutions have also been recently proposed in the context of digital contact tracing, for example, in [7].

Digital contact tracing is an approach that has been built according to the privacy-by-design concept to augment the manual ways of tracing the COVID-19-disease spread. By design, mobile and wireless gadgets equipped with BLE chipsets can transmit and receive anonymized signals with timestamps from nearby devices. This concept has become handy for digital contact-tracing purposes in the past year, since the BLE is a short-range technology that is particularly suitable for estimating close-range distances (e.g., less than 2 m) of the mobile phone users who crossed their paths. The BLE data with temporary identifiers, Received Signal Strength (RSS) values, and the timestamps of the encountered phones are therefore converted into the distance and time spent in proximity. Furthermore, there is a taxonomy [6,8] of centralized and decentralized decision-making approaches to handle data processing and inform the users about the risk of being exposed to the virus.

In the centralized approach [6,9], the logs from the mobile phone (or wearable bracelet) are encrypted and transferred to the cloud with a certain periodicity (e.g., once a day). Therefore in cases where the users opt-in to the protocol, the centralized server estimates the risk of being exposed and conveys this risk to the users. The majority of centralized approaches follow the data minimization principle and request to upload only relevant data, such as the temporary or ephemeral identities of the users who stayed within certain proximity for the time exceeding the set threshold. As an outcome, all computations for the risk scoring are made on the server-side, and the users only receive the notifications.

A different approach, known as decentralized or federated, delegates the risk scoring to own mobile devices or user edge devices, considering the logs are stored locally. Google and Apple adopted the consequent framework in their jointly designed Exposure Notifications protocol described in [10]. Here, only infected users, once confirmed being tested positive, upload the data to the cloud, whereas the rest of the users’ devices download the data from the server and perform the risk estimates locally on their devices. The latter approach assumes that all data shared with the centralized server is subject to the user’s consent.

As subjectively deemed in [6], based on end-user surveys, the users are more likely to perceive the decentralized decision-making approach as a better fit to preserve their location privacy due to the fact that the data is stored locally (typically for up to 21 days, unlike the server-side storage which can be much longer). However, there is no significant threat to the users’ sensitive information in the centralized approach where the logs are encrypted and securely saved on a trusted server. The above-mentioned digital contact-tracing example demonstrates that the location privacy concerns take place in the context of sensitive information, such as one’s whereabouts and identities of encountered contacts.

Location Privacy-Preserving Mechanisms (LPPM) intend to preserve the individual location privacy in scenarios where services request access to the users’ spatial location [11]. Location-Based Services (LBS) that collect sensitive information of the users’ locations, as described in the classification framework in [12], can benefit from implementing LPPM.

Other examples of proximity-based services are ’find-a-friend’ applications [13] or other social-networking applications [14].

In all these proximity-based services, the utility of the services comes from a good detection probability (i.e., the probability to correctly detect two users in the vicinity of each other when they are neighbours, also known as sensitivity measure) as well as a low false-alarm probability (i.e., the probability of incorrectly detecting two users in the vicinity of each other when in fact they are far away). This utility is inherently in a tradeoff with the amount of location privacy that a user can have when disclosing his location.

In order to protect users’ location privacy, many approaches have been proposed so far in the literature. For example, a comprehensive survey of location-privacy mechanisms has been recently provided in [15]. The authors in [15] divided the location-privacy mechanisms into three classes: the Geo-indistinguishability (GeoInd) class, the Local Differential Privacy (LDP) class, and private spatial-decomposition class. They also pointed out that the LDP mechanism is not directly applicable to location data, while the private spatial decomposition requires the presence of a trusted server.

Once LPPM have been implemented, it is necessary to evaluate their behavior and compare it with the initial state of the system. GeoInd refers to a privacy notion that preserves the user’s precise location while revealing approximate geospatial area [16]. Furthermore, when a user disclose its location with a certain perturbation mechanism, this perturbation mechanism can yield GeoInd [17] if the traces of the user are disclosed with a certain radius and certain statistical distributions, such as when Laplacian or Gaussian random perturbations are applied to modify the true user location. The reported location will not reveal information to an adversary for distinguishing the ground truth location among neighboring devices [18].

The authors in [17], presented GeoInd as a possible notion to quantify privacy. They introduced the radius *r*, which corresponds to the level of privacy and showed that such radius is proportional to the location radius, i.e., the Euclidean distance between the true and perturbed locations. Consequently, the radius is increasing by adding controlled randomized (e.g., Laplacian) noise. The authors have encountered problems of discretization and truncation. In our paper we directly use the Euclidian distance between the true and perturbed locations as a measure of user location privacy and we study its tradeoff with the service utility.

Another location privacy-preserving approach in the literature, which is an adherent of Differential Privacy (DP), is the concept of the Private Spatial Decomposition presented in [19]. Private Spatial Decomposition refers to a gradient privacy-budget allocation scheme. The approach assumes a two-dimensional space and different privacy levels, and it is proved to achieve ϵ-differential privacy.

An additional aspect related to the location privacy is the choice of the privacy metric, which is still not unified in the current literature. Such a privacy metric serves to quantify the efficiency of a localization algorithm by exploring the privacy versus accuracy [20] or the privacy versus utility [21] tradeoffs. As above-mentioned, in this paper we measure the location privacy via the Root Mean Square Error (RMSE) between the perturbed location and the true user location.

The authors in [22] proposed a location-aware perturbation scheme for mobile environments, where the goal was to decrease the adversary’s knowledge with added Laplacian noise. Using the Hilbert curve, each second location is projected on a map, thus reducing the overhead caused by the precision of the location estimates. To evaluate the performance and accuracy of the proposed algorithm, the authors in [22] used nearness, resemblance, and displacement metrics. As a common rule, lower levels of ϵ correspond to a higher privacy budget and effectively lower accuracy. For example, in [22], when the ϵ value reached 1.0, the number of points located within 1000 m of the actual positions were a high as 99.04 percent.

Albeit obfuscation mechanisms are growing in their popularity, they introduce errors to the localization system by altering the ground truth locations of the devices. Obfuscation mechanisms result in losing some of the performance, or in other words, the utility of the system. In [18], the authors designed a location obfuscation mechanism, where the GeoInd was satisfied. This work in [18] focused on achieving GeoInd for any pair of neighboring pairs of locations and they showed good results for privacy and utility in 2D spaces. Our work focuses on 3D spaced with multi-floor buildings.

To the best of our knowledge, studies investigating the optimal tradeoff between obfuscating or perturbing the user location (i.e., decreasing the granularity of the reported location) versus utility for proximity-detection applications are still not well explored in the current literature, especially when such a proximity-detection application is a digital contact-tracing solution. Moreover, multidimensional approaches, such as 3D scenarios, provide more freedom for the user to protect their location from an adversary and have not been studied a lot so far.

This paper proposes a new perturbation metric suitable for proximity-detection-based services and applications relying strictly on the relative distance between two users, but not needing absolute location information, offers a theoretical analysis of its properties, and demonstrates via extensive simulation-based results a very good tradeoff between privacy preservation and service utility. The proposed metric is based on a combination of mapping based on the argmax operator and Gaussian or Laplacian perturbations. For comparative purposes, the argmax-based metric is also compared with another metric, based on an argmin operator and Gaussian or Laplacian perturbations, and we show that it has a much better utility-privacy tradeoff than the argmin-based metric. It is to be noticed that the proposed argmax-based metric is only useful in the context of proximity-based services, when only the relative distance between users is needed, but not their absolute location. By contrast, the argmin-based metric would preserve its utility also for other location-based services (in addition to the proximity-based ones), at the expense of lower privacy protection compared to the argmax-based metric.

The remainder of the paper is organized as follows: Section 2 overviews various mechanisms for preserving location privacy in the literature and offers a classification of these mechanisms. Section 3 introduces the two proposed perturbation mechanisms, one based on argmax operator, suitable only for proximity-based services and another one based on argmin operator, suitable for all kinds of location-based services, but with lower privacy preservation levels than the one based on argmax operator. Section 4 offers a mathematical analysis of the proposed argmax operator and proves that it is able to offer GeoInd between users. Section 5 presents detailed simulation results in a 4-floor building with users located both within certain hotspot areas and outside hotspot areas. The presented results are easily scalable to any number of floors. Various configurations, in terms of building size, hotspot density, etc., are analyzed, and detailed results are presented in terms of user privacy and service utility. Finally, Section 6 summarizes the main findings and presents the conclusions.

## 2. Classification of Location-Privacy Mechanisms

**Figure 1:**
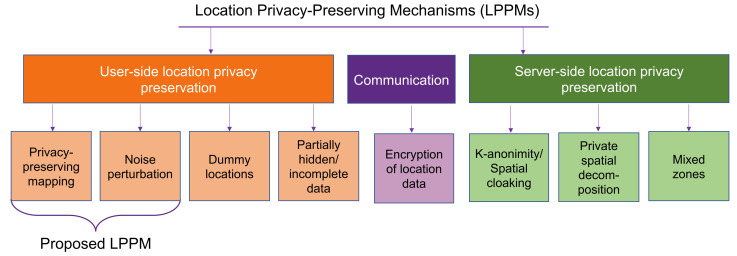
Three-fold classification of location-privacy mechanisms: starting from the edge device, a.k.a. user side (including two parts of the proposed privacy-preserving technique), communication part used for transferring data packets, and server-side perspective including the cases where the users’ data is aggregated on the server.

A classification of location-privacy mechanisms from current literature is provided in Figure 1. The location privacy can be ensured by the server side, by the user side or can be applicable at both sides. A more elaborate explanation of each technique can be found in Table 1 and it is based also on the literature review provided in Section 1.

User-side location privacy mechanisms can be found for example in [23]. Privacy-preserving mappings solutions are born from optimal mappings to preserve privacy against statistical inference [24,25]. Noise perturbation mechanisms based on various noise types, such as Laplace and Gaussian noises are discussed for example in [26,27]. Dummy-location generation has been applied, for example, in [28].

Server-side location privacy mechanisms relying on spatial cloaking and k-anonymity mechanisms are described, for example, in [29,30,31,32]. Unlike in our paper, the assumptions in [32] are that the users communicate their location to the server with high accuracy; in our paper we assume that the users have full control to their location and choose to disclose it to the server with moderate-to-low accuracy, according to the chosen perturbation mechanisms, as explained later, in Section 3.

Private spatial decomposition solutions are discussed for example in [19]. Mix-zones solutions are addressed for example in [33,34]. Secure transformations are conceptually close to the privacy-preserving mappings done at the user/client side and they are addressed for example in [35]. Server-side solutions involve the trust in the service provider and they are susceptible to attacks of the server databases.

A privacy-preserving method that can be applied both at server and user sides is the encryption of location data, via various encryption mechanisms [36,37,38]. Even if encryption/decryption costs are quite affordable by nowadays mobile devices and smartphones, the encryption/decryption studies for location privacy available in the current literature point out that a main drawback of this approach is the relatively high delay [37] introduced in the data encryption/decryption processes, delay which may be not tolerable for many proximity-based services.

Our proposed solutions, described in the next section, is a combination of a privacy-preserving mapping (two mappings provided) and a noisy perturbation (two noise distributions studied).

## 3. Proposed Perturbed Location Mechanism

### 3.1. Scenario Definition, Hypotheses, and Preliminary Notations

We adopt a scenario when user devices are equipped with some form of an indoor localization engine, e.g., a combination of cellular-based positioning, WiFi/BLE-positioning, and other smartphone sensors-based positioning (barometers, gyroscopes, accelerometers), etc., which is already the state-of-the-art of indoor positioning. We also assume that each user *u* can have full control of his/her location data, modeled here via a 3D-location vector $x_{u}\in B$. It is also assumed that the used can choose the perturbation level with which he/she disclose own location data to a service provider. Thus, the user devices are able to apply a local perturbation mechanism $M(x_{u})$, before broadcasting the user location data to a service provider. Such service provider can be, for example, a centralized digital contact-tracing server which computes, based on the available perturbed locations $M(x_{u})$ the relative distances between any two users in the building and compares them to a safety threshold γ (e.g., γ=2 m). The server stores such information in a database, together with timestamps and hashed users identities and when a user *v* informs the server that he or she has been detected with COVID-19, the server is able to find the information about all other users *u* that were in the vicinity of user *v* in a certain time window. For simplicity, we drop the time index in our model and look at snapshot decisions. Thus, if $||M\left(x_{u}\right)-M\left(x_{v}\right)\left|\right|\leq \gamma$, user *u* is informed by the contact-tracing server that he or she has been a ’close contact’. Above, ||·|| is the square root of the Euclidean norm (or the distance between two vectors).

Another example of a service provider relying on such proximity detection is a provider of a ’find a friend’ service. Again, users can install an application which transmits to the service provider the hashed identities of themselves and their friends, and the server is keeping track of the $||M\left(x_{u}\right)-M\left(x_{v}\right)\left|\right|$ distances, based on the perturbed location information transmitted by each user. If $||M\left(x_{u}\right)-M\left(x_{v}\right)\left|\right|\leq \gamma$, then the users *u* and *v* are informed that their friend is nearby, at a distance γ. Again, the threshold parameter γ can be user defined or server defined; most likely, for ’find-a-friend’ application, γ can be higher (e.g., 5–10 m) than for a digital contact-tracing application.

**Figure 2:**
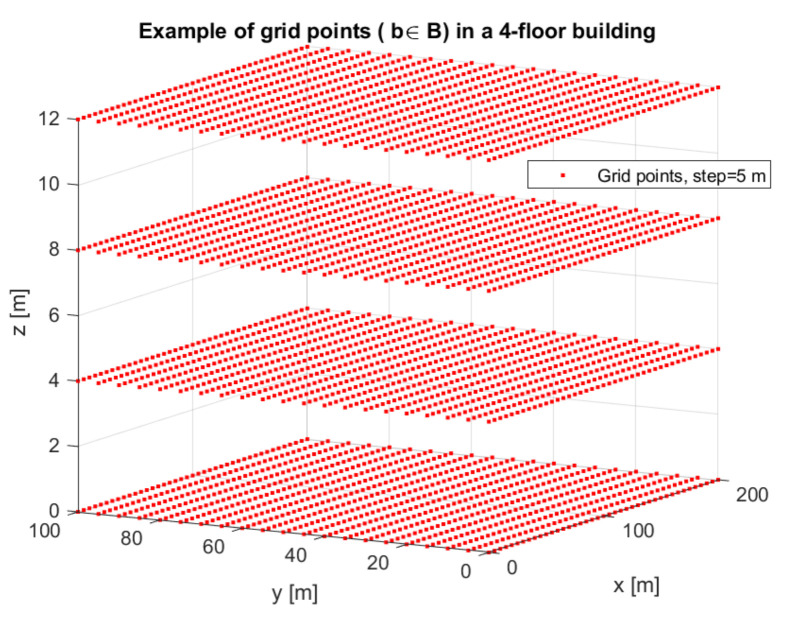
Example of mapping the whole building space B into grid points b, $\Delta_{s}=5$ m for a 100×200$m^{2}$ building with 4 floors and 4 m floor height.

Let us denote the perturbed 3D-location values via $y_{u}$, with $y_{u}=M\left(u_{u}\right)\in B$, with $B\in R^{3}$ being the building space, defined via a cube space with edges $\left[x_{min}\;x_{max}\right]\times \left[y_{min}\;y_{max}\right]\times \left[z_{min}\;z_{max}\right]$, where $x_{min},x_{max},y_{min},y_{max},z_{min},z_{max}$ are the building edges (minimum and maximum, respectively) in the 3D space. It is assumed that the centralized digital contact-tracing server (which can be trusted or untrusted) has access to the building floor plans. It is also assumed that the server is dividing the whole building space into grid points $b=\left[b_{x},b_{y},b_{z}\right]\in B^{3}$, for example as shown in Figure 2 and that the set of grid points {b|b∈B } is transmitted to all users in the building, e.g., via cellular or WiFi connectivity. The grid step $\Delta_{s}$ is a parameter of the centralized server providing proximity-detection services or user digital contact tracing. With a $\Delta_{s}$ step it means that $b_{x}$ for example can only take values in the interval $\left[x_{min}:\Delta_{s}:x_{max}\right]$.

### 3.2. Perturbation Metrics

Two perturbation metrics are proposed and investigated, as defined in Equations (1) and (2).

(1) $$M_{argmin}\left(u_{u}\right)=argmin_{b\in B}||b-x_{u}\left|\right|+\xi$$

where ||·|| is the distance between b and $x_{u}$ vectors and ξ is a multivariate (3D) noise vector of zero mean (to be explained later in this section). Also,

(2) $$M_{argmax}\left(u_{u}\right)=argmax_{b\in B}||b-x_{u}\left|\right|+\xi$$

While the argmin operator is rather intuitive, stating that the user location is only slightly perturbed by mapping it to the nearest grid point and then adding a random noise to it, the argmax operator may seem less intuitive at a first glance. Indeed, with argmax operator, all users located, for example, at the extreme north-west of the building, will be mapped, after argmax operator, as being close to the extreme south-east of the building. As we are only focusing here on the proximity-detection type of application relying on the relative distance between users, such as digital contact tracing or find a friend, this mapping does not decrease the service utility, as nearby users (which were, for example, at the extreme north-west of the building) will still appear as nearby users after the mapping to the other side of the building.

In order for $M_{argmin}\left(u_{u}\right)$ and $M_{argmax}\left(u_{u}\right)$ metrics to remain inside the building space B and to offer plausible perturbed locations, an additional correction is done after the mappings in Equations (1) and (2), in such a way that the points that would fall outside the building edges, are re-mapped to the nearest point inside the building. In addition, if the perturbed *z* coordinate does not match any of the floor heights in the building, then the perturbed *z*-coordinate is mapped to the nearest floor level. Examples will be provided in Section 5.

The *argmin* metric in Equation (1) is mapping the true position to the nearest grid point in the building and it then applies a noise factor to it, while the *argmax* metric in Equation (2) is mapping the true position to the furthest grid point in the building and it then applies a noise factor to it. Clearly, on one hand, Equation (1) mapping preserves a minimum distance between the perturbed location and the true location, enabling various location-based services that require absolute user-location knowledge, but it acts quite poorly in terms of privacy preservation, as an attacker could still identify the approximate location of an user with an accuracy depending on the inverse of the standard deviation 1/ϵ of the added multivariate noise ξ. On the other hand, the second proposed metric from Equation (2) is able to protect the user location privacy to a great extent (as the privacy increases when the distance between the perturbed location and original location increases), with an increased privacy level for larger/wider buildings, and, as we will show in Section 5, without destroying the usefulness of the services, meaning that an accurate contact tracing can be also achieved under a heavy protection of user’s location privacy.

Regarding the added noise vector ξ, two multivariate noise distributions are considered, namely a Gaussian distribution of equal standard deviation in x,y,z dimensions of 1/ϵ, see Equation (3), and a Laplacian distribution of equal scale factor in x,y,z dimensions of 1/ϵ, see Equation (4). The zero-mean multivariate (3D) Gaussian noise is:

(3) $$f_{Gauss}\left(\xi \right)=\frac{1}{{\left(2\pi \right)}^{1.5}{\left|\Sigma \right|}^{0.5}}exp\left(-0.5\xi^{T}\Sigma^{-1}\xi \right)$$

with $\Sigma =diag\left(\left[\frac{1}{\epsilon }\;\frac{1}{\epsilon }\;\frac{1}{\epsilon }\right]\right)=\frac{1}{\epsilon }I_{3}$ being a diagonal covariance matrix and $I_{3}$ a unit matrix of dimension 3×3, and $\left|\Sigma \right|=\epsilon^{-3}$ being the determinant of Σ.

The zero-mean multivariate (3D) Laplacian noise is:

(4) $$f_{Laplace}\left(\xi \right)=\frac{2}{{\left(2\pi \right)}^{1.5}{\left|\Sigma \right|}^{0.5}}{\left(0.5\xi^{T}\Sigma^{-1}\xi \right)}^{-0.5}K_{v}\left(\sqrt{2\xi^{T}\Sigma^{-1}\xi }\right)$$

where $K_{v}$ is the modified Bessel function of second kind.

### 3.3. Private Proximity-Detection Architecture with the Proposed Mechanism

**Figure 3:**
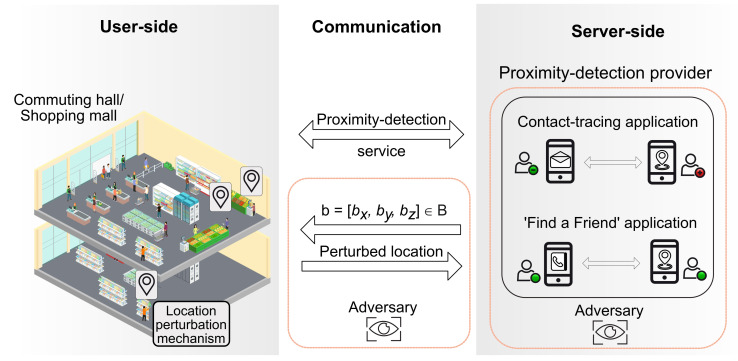
An illustration of the considered scenario: a building (e.g., a shopping mall) with users willing to use the digital contact-tracing and/or ‘find-a-friend’ applications. The ’Adversary’ entity refers to any third party which aims to access the information about devices’ whereabouts.

The wireless communication process between user/edge devices and the proximity-detection service is depicted in Figure 3. Users are assumed to be spread across a multi-floor space of commercial or commuting interest (e.g., shopping mall, commuting hall/airport/ train station, etc.). Users’ devices are supposed to be equipped with a localization engine, such as GNSS, WiFi, BLE or a combination of several localization methods. A proximity service provider is operating in the building of interest, with access to the building floor plans and able to send the floor-map coordinates **b** to all users interested in the proximity-based service or application. The coordinates can be provided as Earth Centered Earth Fixed (ECEF) coordinates, as (latitude, longitude, and altitude)-coordinates, or as local coordinates (x,y,z) and the mapping between any of these coordinate systems is assumed known both at the user side and at the server side. The user devices performs the location perturbation locally and sends the perturbed location to the server; the server processes in an aggregate form all the data based on the perturbed locations of the users inside the building and offers the proximity-based service to the users.

## 4. Theoretical Analysis of the Proposed Argmax Perturbed Location Mechanism

For simplicity, in this section we focus on the argmax metric from Equation (2) and we denote via $M\left(\cdot \right)=M_{argmax}\left(\cdot \right)$, with the observation that similar derivations can be obtained in a straightforward manner for argmin metric. Let denote by $p_{u}$ the probability that an adversary finds out $x_{u}$ by listening to $y_{u}=M\left(x_{u}\right)$. Then

(5) $$\begin{array}{c} p_{u}=proba\left(M\left(x_{u}\right)=x_{u}\right)=proba(argmax_{b\in B}||b-x_{u}\left|\right|+\xi =x_{u})\\ =proba(\xi =x_{u}-argmax_{b\in B}||b-x_{u}||) \end{array}$$

If we denote via $a_{u}≜argmax_{b\in B}||b-x_{u}\left|\right|$, under Gaussian-noise assumption, the above formula is determined by the Gaussian noise probability distribution function (PDF) from Equation (3) and it becomes equal to

(6) $$\begin{array}{c} p_{u}=\frac{\epsilon^{3}}{{\left(2\pi \right)}^{1.5}}exp(-0.5\epsilon ||x_{u}-a_{u}{\left|\right|}^{2}) \end{array}$$

Similarly, if $p_{v}$ is the probability that an adversary intercepts the perturbed location of user *v*, namely $M_{argmax}\left(x_{v}\right)$ and maps it to the location of user *u*, after straightforward derivations (as above) and following the Gaussian noise assumption, we get

(7) $$\begin{array}{c} p_{v}=\frac{\epsilon^{3}}{{\left(2\pi \right)}^{1.5}}exp(-0.5\epsilon ||x_{u}-a_{v}{\left|\right|}^{2}) \end{array}$$

with $a_{v}≜argmax_{b\in B}||b-x_{v}\left|\right|$.

By dividing Equation (6) to Equation (7) and using Cauchy-Schwarz inequality, one gets

(8) $$\begin{array}{c} \frac{p_{u}}{p_{v}}=exp\left(0.5\epsilon \left(\left|\right|x_{u}-a_{u}{\left|\right|}^{2}-\left|\right|x_{u}-a_{u}{\left|\right|}^{2}\right)\right)\\ \leq exp\left(0.5\epsilon ||a_{u}-a_{v}{\left|\right|}^{2}\right)\\ \leq exp\left(0.5\epsilon ||x_{u}-x_{v}{\left|\right|}^{2}\right) \end{array}$$

Thus, the proposed mechanism M(·) offers GeoInd type of user location privacy.

## 5. Simulation-Based Results

### 5.1. Simulation Scenarios and Performance Metrics

A 4-floor scenario with $N_{u}$ users spread within the building, with most of them within couple of pre-defined hotspot areas was considered. Table 2 shows the main parameters used in the simulation model (additional parameters were investigated in some scenarios and they are specified in the figures’ captions when different from those in Table 2). The users are assumed to transmit their perturbed location $M(x_{u})$ to a server provider offering a proximity-based service with a proximity threshold γ (i.e, the service is offered if the users are determined to be at a distance less than γ, based on their perturbed location transmitted to the server).

**Figure 4:**
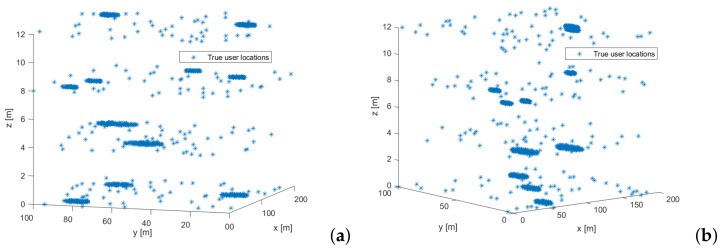
Two examples of users distribution within a 4-floor building during two Monte Carlo runs. (**a**) Monte Carlo run 1; (**b**) Monte Carlo run 2. In these runs, we allocated 80% of users are in hotspot areas and 20% of users are outside hotspot areas, uniformly distributed within the building.

At each Monte Carlo run, another realization of users’ random positions within the building is implemented. Two examples of the users distribution in the building during two Monte Carlo runs is shown in Figure 4.

**Figure 5:**
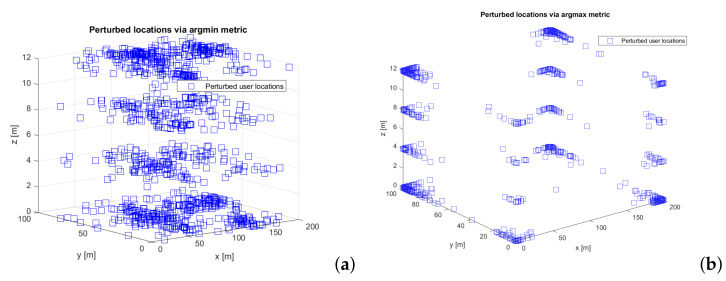
Examples of perturbed locations based on (**a**) $M_{argmin}\left(\cdot \right)$ and (**b**) $M_{argmax}\left(\cdot \right)$ metrics. ϵ=0.1 m, Laplace perturbation.

Examples of perturbed locations during one Monte Carlo run with *argmin* metric (left plot) and *argmax* metric (right plot) are shown in Figure 5, for ϵ=0.1 and Laplacian noise.

**Figure 6:**
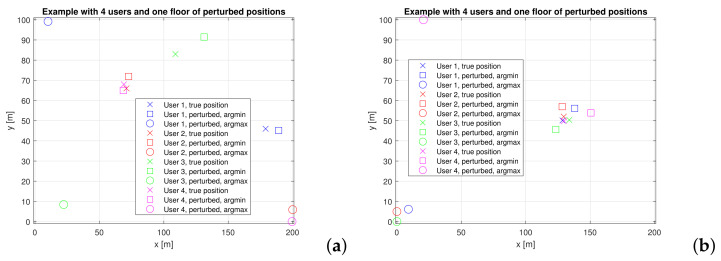
Two examples of perturbed location via argmin + Laplacian noise and via argmax + Laplacian noise. (**a**) users uniformly distributed over one floor; (**b**) users uniformly distributed within a circular hotspot of radius 5 m.

A zoomed version of perturbed locations for one floor and with only 4 users is illustrated in Figure 6, this time showing both the scenario with no hotspots (left plot) and with hotspots (right plot). The squares show the perturbed location via *argmin* metric and the circles show the perturbed location via *argmax* metric.

The utility functions are defined as the probability of correctly detecting two users to be in close proximity to each other $P_{d}$, as well as the complement of the false alarm probability $P_{fa}$, meaning the probability to detect that two users are in close proximity to each other, when in fact they are not. Mathematically, $P_{d}$ and $P_{fa}$ are defined via

(9) $$P_{d}=\frac{|\{\left(u,v\right)\in N_{u}\times N_{u},u\neq v\;|\;∥M\left(x_{u}\right)-M\left(x_{v}\right)∥\leq \gamma \;and\;∥x_{u}-x_{v}∥\leq \gamma \}|}{|\{\left(u,v\right)\in N_{u}\times N_{u},u\neq v\;|\;∥x_{u}-x_{v}∥\leq \gamma \}|}$$

and, respectively,

(10) $$P_{fa}=\frac{|\{\left(u,v\right)\in N_{u}\times N_{u},u\neq v\;|\;∥M\left(x_{u}\right)-M\left(x_{v}\right)∥\leq \gamma \;and\;∥x_{u}-x_{v}∥\geq \gamma \}|}{|\{\left(u,v\right)\in N_{u}\times N_{u},u\neq v\;|\;∥x_{u}-x_{v}∥\geq \gamma \}|}$$

where |·| is the cardinal operator, $N_{u}$ is the number of users inside the building, and $P_{d}$ and $P_{fa}$ correspond to detection probability (here also the sensitivity) and false positive rate in confusion-matrix terminology, respectively. Clearly, the proximity-based service utility increases when $P_{d}$ increases and when $P_{fa}$ decreases.

The ensured privacy level is proportional to the distance between the perturbed location and the true location, or the RMSE between $M(x_{u}$) and $x_{u}$, namely

(11) $$RMSE=\sqrt{\frac{1}{N_{u}}\sum_{u=1}^{N_{u}}||M\left(x_{u}\right)-x_{u}{\left|\right|}^{2}}$$

Clearly, the ensured privacy level is better when RMSE from Equation (11) is higher.

### 5.2. Comparison with State-of-the-Art Perturbation Mechanisms

Several obfuscation models have been proposed so far in the literature to protect the location information, as described in Section 2. Three of the most common ones, selected here as benchmarks are the uniform obfuscation [31], the Laplacian perturbation [47], and the Gaussian perturbation [48]. The uniform perturbation model from [31] was given for 2D case and it was based on the idea that a random vector shift is applied to the user location with a certain radius. The model from [31] extended to 3D scenarios can be written as

(12) $$M_{uniform}\left(u_{u}\right)=x_{u}+\xi_{u}$$

where $\xi_{u}$ is a 3D vector with elements $\left[\xi_{u,x},\xi_{u,y},\xi_{u,z}\right]$ given by

(13) $$\begin{array}{c} \xi_{u,x}=\mu cos\left(\theta \right) \end{array}$$

(14) $$\begin{array}{c} \xi_{u,y}=\mu sin\left(\theta \right) \end{array}$$

(15) $$\begin{array}{c} \xi_{u,z}=\mu tan\left(\alpha \right) \end{array}$$

and μ, θ, and α are the random radius, azimuth, and elevation angles, respectively, drawn from the following three uniform distributions: μU(0,1/ϵ), θU(0,2π), and αU(0,2π), where U(a,b) stands for a uniform distribution in the interval [a,b].

**Figure 7:**
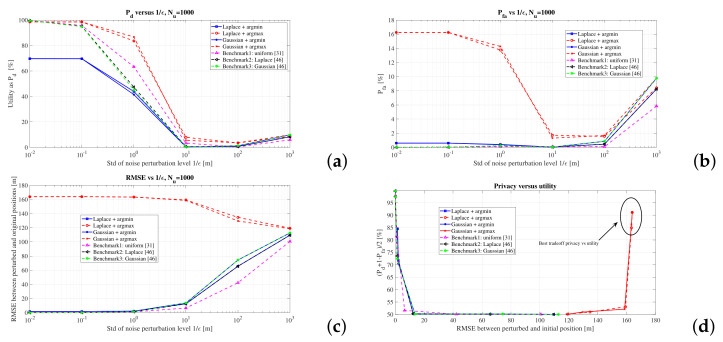
Comparison with state-of-the-art algorithms: (**a**) $P_{d}$ versus the noise perturbation level; (**b**) $P_{fa}$ versus the noise perturbation level; (**c**) RMSE between the perturbed location and original location versus the noise perturbation level; (**d**) utility versus privacy.

The Laplacian [47] and Gaussian [48] perturbations can be modeled as

(16) $$M_{Laplace,Gaussian}\left(u_{u}\right)=x_{u}+\xi$$

where ξ is a Laplacian or a Gaussian noise, as given in Equations (4) and (3), respectively. The comparison with the three state-of-the-art algorithms described above, namely uniform obfuscation [31], Laplacian perturbation [47], and Gaussian perturbation [48] is shown in Figure 7.

As seen in Figure 7, the argmax-based metric offers the best detection probability (upper left plot) and the best privacy level (lower left plot), but slightly worse false alarm probabilities (upper right plot) than the other four investigated algorithms, namely argmin-based and three bench, ark ones. The most important plot is however the one depicted in the lower right part of Figure 7, where the utility-privacy tradeoff is illustrated. For a fairer comparison, the utility here comprises the average between the $P_{d}$ and $1-P_{fa}$; the closest to 100% this value is, the higher utility we have; ideally, a best service would have $P_{d}=1$ and $P_{fa}=0$. The privacy level is given by RMSE; the higher the RMSE between the perturbed and true location is, the higher the privacy. Clearly, the argmax-based perturbation is a clear winner among all considered algorithms, as it can reach simultaneously high levels of privacy and high levels of utility of a proximity service relying in inter-users distance. It is to be emphasized that such utility pertains only to such proximity-based services relying on inter-user distances; other location-based services needing absolute location information would have a different utility, where our argmax-based algorithm would most likely perform poorer than the other approaches. In terms of argmin-based approach versus the three considered benchmark, there is very little difference in the utility-privacy tradeoff. For this reason and in order to keep clarity in the subsequent plots, we will focus from now on only on the comparisons between argmin- and argmax-based perturbations and on the deeper analysis of the argmax-based operator.

### 5.3. Privacy Level as a Function of ϵ Parameter

**Figure 8:**
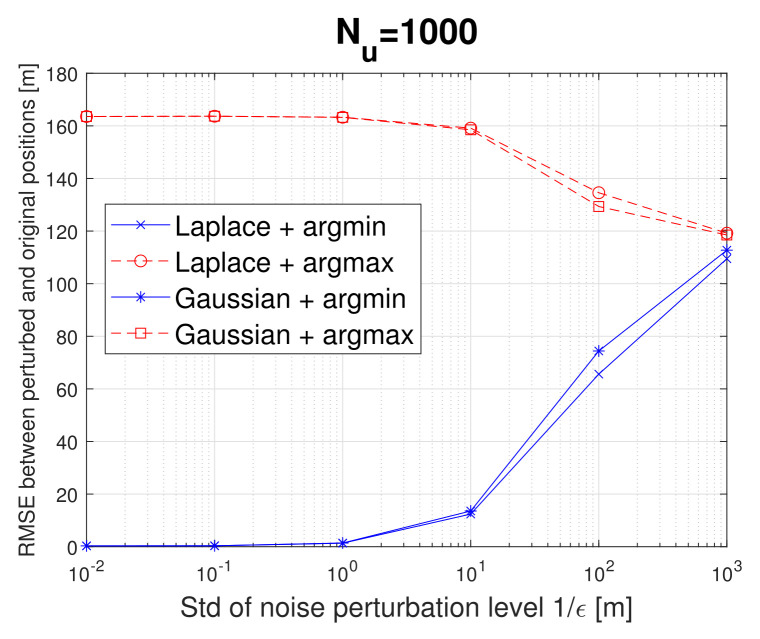
RMSE between the perturbed location and original location versus the noise perturbation level for two noise types (Laplacian and Gaussian) and two mapping metrics (argmin and argmax).

The RMSE between the transmitted perturbed location and the original location, as defined in Equation (11), is shown in Figure 8. A higher RMSE value means a higher user privacy level. There is no significant difference between the noise type ξ used in the perturbation mechanism, with the Laplacian noise giving slightly better results than the Gaussian one in terms of privacy for the *argmax* metric, and the Gaussian noise giving slightly better results in terms of privacy for the *argmin* metric.

A very interesting finding is that by using an *argmax* metric, not only one achieves significantly higher privacy level than by using *argmin* metric (i.e., higher RMSE values), but also the noise level 1/ϵ acts in an opposite manner on the *argmax* metric than on the *argmin* metric, meaning that a higher ϵ ensures more obfuscation in the argmin-based approach, but less obfuscation in the argmax-based approach. This points out that high levels of ϵ (or, equivalently low levels of the noise standard deviation) are giving better results in terms of privacy with the *argmax* metric than lower levels of ϵ. This is observed due to the fact that the users’ location is already mapped far away from its initial location through the *argmax* operator, and it is enough to add only a small additional random perturbation in order to make difficult the ’guessing’ of true user location $x_{u}$ based on the disclosed perturbed location $M(x_{u})$ in case an attacker or eavesdropper gets access to the perturbed location.

### 5.4. Utility Level as a Function of ϵ Parameter

**Figure 9:**
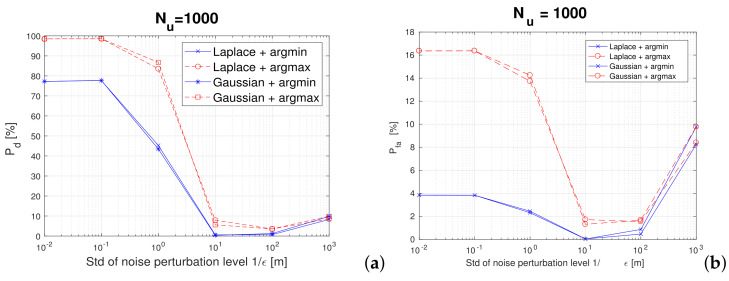
(**a**) Detection and (**b**) false-alarm probabilities versus the noise perturbation level for two noise types (Laplacian and Gaussian) and two mapping metrics (argmin and argmax). The proximity threshold γ was set to 2 m (e.g., for a digital contract-tracing application). A 4-floor building with 1000 users and 80% of them placed in hotspot areas.

Figure 9 shows the utility (i.e., the detection probability) as well as the false alarm probabilities in the presence of various perturbations (*argmin* versus *argmax* and Gaussian versus Laplacian noises).

Clearly, the *argmax* metric has higher utility at the expense of a moderately higher false alarm than the *argmin* metric. The differences between Gaussian and Laplacian noises are minor and therefore Gaussian perturbation is recommended to be used for simplicity. The best detection probabilities for a proximity-based application are achieved with ϵ values above 1 (or equivalently, standard deviation of the noise below 1 m). We can see from the left plot in Figure 9 that detection probabilities close to 100% are achievable with the proposed *argmax* metric, with moderate false alarms of about 16%. As the user privacy is highly preserved with an *argmax* metric and high enough ϵ values (see also Figure 8), the price to pay in terms of false alarm probabilities of up to 16% may seem reasonable for users desiring high location privacy. Indeed, the cost of a false alarm may be quite low to the user (e.g., user is incorrectly informed that a friend is nearby or user is incorrectly informed that he or she might have been close contact of a person confirmed with COVID-19 and thus he/she would take unnecessary, but also not-hurtful additional protection measures). However, the utility of a correct proximity detection in a proximity-based service is high and, as shown in the left plot of Figure 9, it is preserved with the $M_{argmax}$ metric and an ϵ value above 1.

### 5.5. Privacy-versus-Utility Tradeoffs

**Figure 10:**
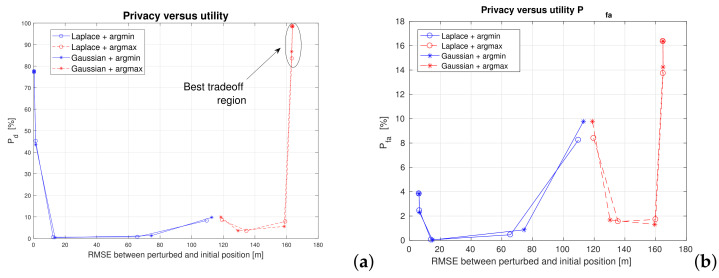
Privacy versus utility tradeoff. Proximity threshold γ=2. The plots illustrate the behavior of the argmin vs argmax metrics against RMSE. (**a**) $P_{d}$ as utility; (**b**) $P_{fa}$ as utility

An illustration of the privacy-versus-utility tradeoff is shown in Figure 10, where the utility is defined as the correct detection probability $P_{d}$ (see Equation (9)).

**Figure 11:**
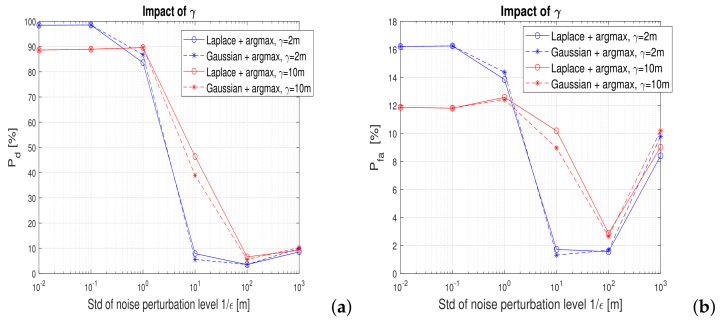
Impact of the proximity threshold on (**a**) detection $P_{d}$ and (**b**) false-alarm rates $P_{fa}$.

**Figure 12:**
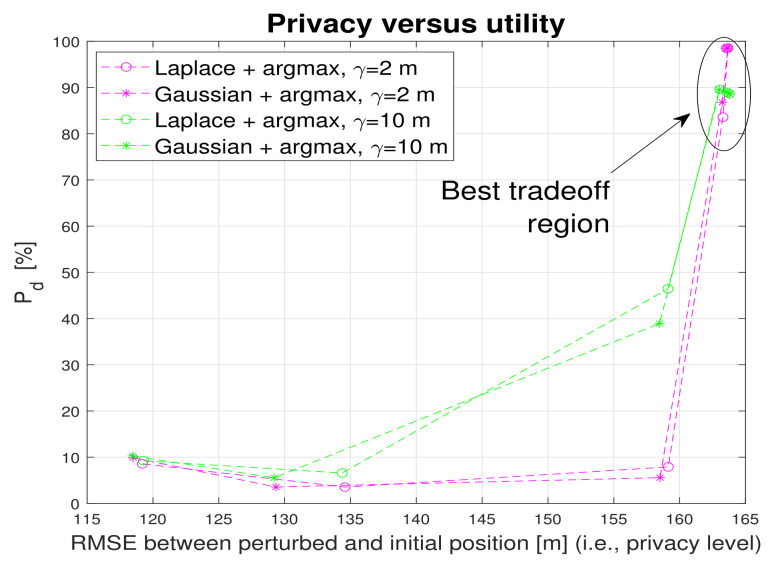
Privacy versus utility tradeoff. Argmax metric. Proximity thresholds γ=2 m and γ=10 m.

Figure 11 shows also the impact of the proximity threshold γ on the utility (detection probability) and false alarm probability. Two proximity thresholds were considered: γ=2 m, useful for example for a digital contact-tracing service provider and γ=10 m, useful for example for a ’find a friend’ application in a shopping center. The proximity threshold choice does not change the main conclusions that *argmax* metric with an ϵ below 1 (i.e., a noise standard deviation above 1 m) offers the best tradeoff between utility and privacy. This threshold provides decent detection probabilities (higher than 90%) and moderately low false alarm probabilities (below 16%). The best tradeoff utility region is also illustrated in Figure 12, this time only for the *argmax* metric and two proximity thresholds.

**Figure 13:**
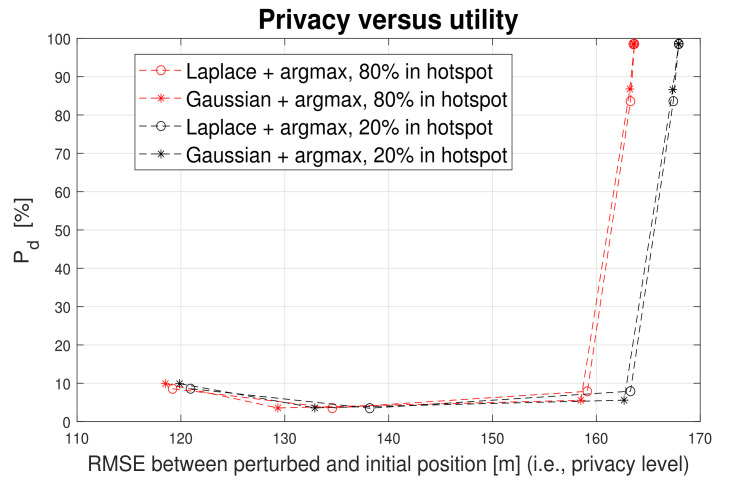
Privacy versus utility tradeoff in the presence of different hotspot distribution of users (80% of users within hostpots versus only 20% of users within the building hotspots). Argmax metric and γ=2 m.

Figure 13 shows that also the hotspot distribution of users has little bearing on the privacy-utility tradeoff, with best tradeoffs obtained again for *argmax* metric and a low ϵ value, mapping to high perturbed levels due to *argmax* operator. As in the $M_{argmax}\left(\cdot \right)$ metric, the user perturbed location is mapped to points far away from true user location, it is intuitive that higher RMSE values between the perturbed and true locations are obtained in the case with less users within the building hotspots, as seen in Figure 13 by comparing the 20% and 80% hotspot distributions.

**Figure 14:**
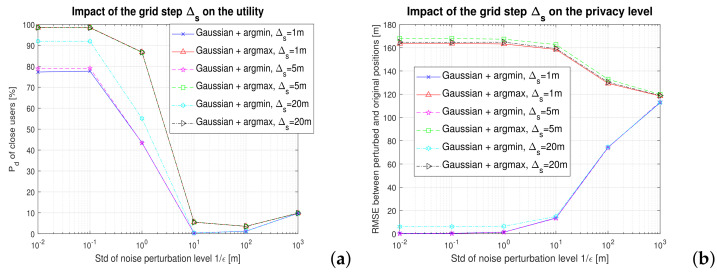
The impact of the grid step on the (**a**) utility and (**b**) privacy. A proximity service with γ=2 m.

The impact of the grid step on the utility and the privacy level is shown in Figure 14. As mentioned above, the grid step influences the matrix b∈B transmitted to the users within a building. For clarity purpose and because the noise type (Laplace versus Gaussian) has low impact, only the Gaussian noise perturbations are shown. Clearly, the impact of the step size is minimal on both the service utility (computed as the correct detection probability of close-by users within a threshold γ) and on the user privacy (computed as the RMSE between the disclosed perturbed location and the true user location). This fact eases the amount of data needed to be transferred from the service provider to the user, as the size of the building grid matrix b is decreasing when the grid step $\Delta_{s}$ is increasing. Nevertheless, the choice of the grid step $\Delta_{s}$ should take into account the building size (e.g., steps lower than 10% of maximum building length in a certain direction are recommended).

**Figure 15:**
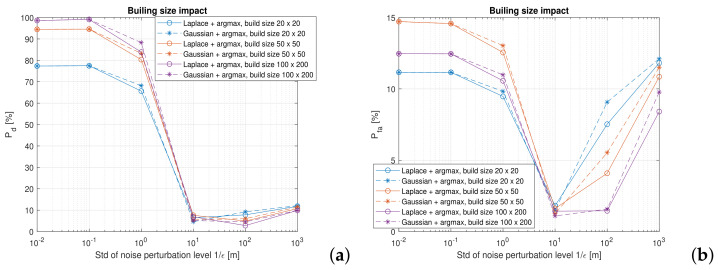
The impact of the building size on the application’s utility. A proximity service with γ=2 m, fixed $N_{u}=1000$. (**a**) $P_{d}$ and (**b**) $P_{fa}$.

In Figure 15, the different building sizes are compared for a fixed number of users $N_{u}$. Here, the added noise in the perturbation yields similar results independent of its type. However, $P_{d}$ levels are high up, as close to 100% for the largest building size, namely 20 × 20 m. Whereas the smallest building considered in the simulation, with the dimensions of 100 × 200 m, shows moderate $P_{d}$ and $P_{fa}$ levels, accordingly. One could translate the situation with a fixed number of users and varying building sizes into the density of the users, where a little space is offered to each user per se.

**Figure 16:**
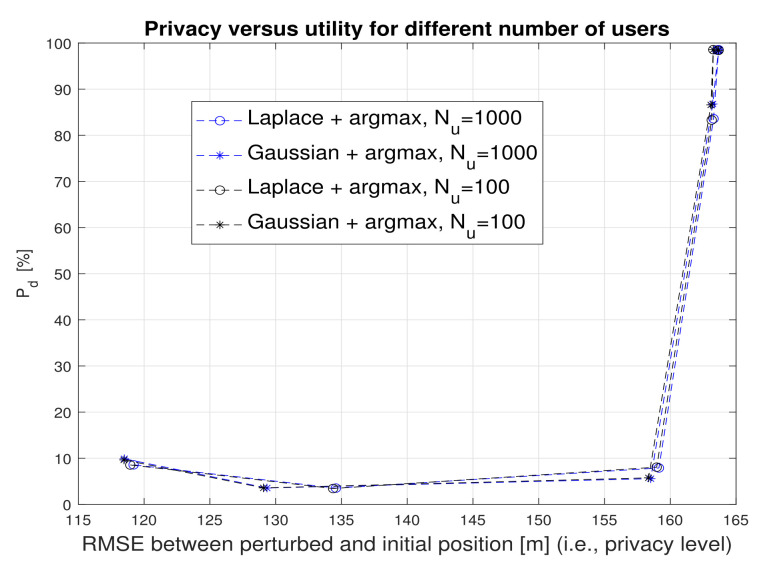
Privacy versus utility tradeoff in the presence of different number of users. Argmax metric and γ=2 m.

Last but not least, Figure 16 shows that the number of users in the building has no impact on the utility-privacy tradeoff and the *argmax* metric with any of the two noise types (Gaussian or Laplacian) is able to attain very good tradeoff levels.

## 6. Conclusions

This paper has proposed a local perturbation mechanism for preserving user-location privacy, while maintaining a high utility of proximity-detection-based services such as digital contact tracing or find-a-friend application. We would like to emphasize that the proposed argmax-based mechanism is useful only for applications relying strictly on the relative distance between any two users, such as digital contact tracing. However, the system loses its utility in the context of location-based services requiring absolute user location, such as finding the nearest shop or searching for a specific route in a mall.

The proposed mechanism is able to offer GeoInd and a very good privacy-utility tradeoff. It relies on the assumption that users have full control of the disclosure level of their location accuracy. Moreover, it is assumed that the service provider has access to the floor plans of the buildings of interests (e.g., a commuting hall, a shopping mall, etc.) and is transmitting the discretized grid map (in terms of x, y, z coordinates) of the building. to all users in the building.

We have provided detailed simulation-based results in a multi-floor building scenario, under different assumptions of user location distributions, grid map step size, hotspot distributions, and number of users in the building. We have also compared the proposed *argmax*-based metric with an *argmin*-based metric and other state-of-the-art metrics which would be useful in location-based services requiring absolute location information, not only relative location information as needed in proximity-based services. We have shown that argmax-based approach with a perturbation level 1/ϵ between 1 and 10 cm offers the best tradeoff utility-privacy for proximity-based services, while argmin-based metric is more suitable for services requiring absolute location information. We have also shown that the number and distribution of users in a building, the random distribution type (Gaussian or Laplacian), as well as the building grid steps have little impact on the results. We were able to reach, via the argmax-based mechanisms, very good privacy levels (RMSE in the orders of the building sizes) with detection probabilities of the order of 90% and false alarm probabilities below 15%. The simulations have also shown that the service utility, measured as detection probability, which is slightly better for large buildings and low γ threshold than for small buildings and high γ threshold. At the same time, the false alarm probabilities are slightly better for small buildings and high γ threshold than for large buildings and low γ threshold. The γ threshold is highly dependent of the target proximity-based service (e.g., we considered γ=2 m for digital contact-tracing applications and γ=10 m for ’find-a-friend’ type of applications).

Open challenges are related to mechanisms for ensuring full user control on local devices about his/her/their location information, the impact of the imperfect knowledge of the user location information (or true position), as well as the impact of imperfect floor-map knowledge (e.g., incorrect floor heights) from the proximity service provider’s point of view.

## Figures and Tables

**Table 1:** Overview of LPPM in the literature.

Location-Preservation Area	Mechanism	Main Features	Refs.
User-side	Privacy-preserving mapping	Multiple initialization and data collection steps are required to build the initial map for further feature extraction and matching.	[24,25]
User-side	Noise Perturbation	The concept of adding noise from a sample distribution and modifying the reported locations of the users. This approach is easy to break in cases where the adversary has prior knowledge about the noise model in use.	[26,27,39]
User-side	Dummy locations	The mechanism is susceptible to inference attacks, easy to break with an application of heterogeneous location correlations.	[28,40,41]
User-side	Partially hidden (incomplete) data	This method assumes ditching or deliberately hiding non-essential pieces of data, which could reveal sensitive information of the users’ whereabouts. This method is easy to break with an application of heterogeneous correlations.	[39]
Communication	Encryption	For security reasons, all data should be encrypted, consequently, this might cause insignificant delays in transferring the packets within a communication scheme [42].	[36,37,38]
Server-side	k-anonymity/ Spatial cloaking	Minimizes risks of re-identification of anonymized data; however, this approach is susceptible to privacy breaches, such as de-anonymization, in cases where the adversary has prior knowledge about individuals. To tackle the issue, such approaches as *t-closeness* and *l-diversity* were developed to augment the *k-anonymity* privacy protection [43,44].	[29,45]
Server-side	Private spatial decomposition	Via applications of the hierarchical decomposition, the location data is stored in clusters, being decomposed into small pieces.	[19,46]
Server-side	Mixed zones	This method aggregates the user data with common attributes and generalizes the location to set areas, having bigger radii than the ground truth location. Therefore, it is not providing a solid basis for preserving privacy as some data are still revealed.	[33,34]

**Table 2:** Main simulation parameters (unless otherwise specified in plots’ titles).

Parameter	Value [Unit]
Number of floors $N_{f}$	4 [-]
Building grid $\Delta_{s}$	1 [m]
Building size	100×200 [m${}^{2}$] horizontally
	12 m vertically (4 m floor heights)
Number of users $N_{u}$	Variable, 100 or 1000 [-]
Privacy budget ϵ	Variable, between $10^{3}$ and $10^{2}$ [1/m]
Proximity threshold γ	Variable, 2 or 10 [m]
Number of hotspots per floor	Variable, between 2 and 4 [-]
Hotspot radius	Variable, between 4 and 10 [m]
Percentage of users within hotspot areas	80[%]
Number of Monte Carlo runs	1000 [-]

## List of Acronyms

6G	Sixth generation of cellular communications
BLE	Bluetooth Low Energy
COVID-19	Coronavirus disease 2019
DP	Differential Privacy
ECEF	Earth Centered Earth Fixed
GNSS	Global Navigation Satellite Systems
GeoInd	Geo-indistinguishability
IEEE	Institute of Electrical and Electronics Engineers
LDP	Local Differential Privacy
LBS	Location-Based Services
LPPM	Location Privacy-Preserving Mechanisms
PDF	probability distribution function
RSS	Received Signal Strength
RMSE	Root Mean Square Error
UWB	Ultra Wide-Band
